# Tissue Distribution and Penetration of Isavuconazole at the Site of Infection in Experimental Invasive Aspergillosis in Mice with Underlying Chronic Granulomatous Disease

**DOI:** 10.1128/AAC.00524-19

**Published:** 2019-05-23

**Authors:** Annie Lee, Brendan Prideaux, Min Hee Lee, Matthew Zimmerman, Enriko Dolgov, David S. Perlin, Yanan Zhao

**Affiliations:** a Public Health Research Institute, Rutgers Biomedical and Health Sciences, Newark, New Jersey, USA; b Department of Neuroscience, Cell Biology, and Anatomy, University of Texas Medical Branch, Galveston, Texas, USA

**Keywords:** chronic granulomatous disease, drug penetration, invasive aspergillosis, isavuconazole, isavuconazonium sulfate, laser capture microdissection, matrix-assisted laser desorption ionization mass spectrometry imaging

## Abstract

Isavuconazole, the active moiety of the prodrug isavuconazonium sulfate, has potent activity against a wide spectrum of fungal pathogens and is approved for the treatment of invasive aspergillosis, yet little is known about the tissue penetration of isavuconazole at the target sites of infection. Here, we explored the spatial and quantitative distribution of isavuconazole in tissue lesions in experimental pulmonary aspergillosis established in mice with chronic granulomatous disease (CGD) (gp91^phox−^). Matrix-assisted laser desorption ionization mass spectrometry imaging (MALDI-MSI) and laser capture microdissection (LCM)-directed high-pressure liquid chromatography coupled to tandem mass spectrometry (LC-MS/MS) were used to analyze infected lungs and brain tissues collected 1, 3, 6, and 24 h after a single oral administration of the prodrug at a dose of 256 mg/kg of body weight (corresponding to 122.9 mg/kg of isavuconazole). Drug enrichment within granulomatous lesions was observed in lung tissue at 1 h postdose, although drug levels quickly equilibrated afterwards between lesion and nonlesion areas. A prominent antifungal effect in the infected lung tissue was revealed by histopathological analysis. Isavuconazole also penetrated into the brain with high efficiency. These data further support the value of isavuconazole to treat patients with invasive aspergillosis.

## Abstract (precis)

Isavuconazole, the active moiety of the prodrug isavuconazonium sulfate, has potent activity against a wide spectrum of fungal pathogens and is approved for the treatment of invasive aspergillosis, yet little is known about the tissue penetration of isavuconazole at the target sites of infection. Here, we explored the spatial and quantitative distribution of isavuconazole in tissue lesions in experimental pulmonary aspergillosis established in mice with chronic granulomatous disease (CGD) (gp91^phox−^).

## INTRODUCTION

Invasive aspergillosis (IA) is a devastating infection and a common cause of death for the immunocompromised patient population (1–3). The development of newer *Aspergillus*-active triazoles after itraconazole was a major step forward in IA therapy. Voriconazole is currently recommended as a first-choice treatment for IA, and posaconazole is indicated for prophylaxis and salvage therapy (4, 5). However, morbidity and mortality rates of IA still remain unacceptably high (5), and clinical management has been further complicated due to the emergence of azole resistance in Aspergillus fumigatus (6, 7). Therefore, alternative treatment regimens and the development of novel compounds are urgently needed.

Isavuconazole, the active moiety of the novel prodrug triazole compound isavuconazonium sulfate, has potent activity against a wide spectrum of fungal pathogens, including *Aspergillus* species (8, 9). The prodrug isavuconazonium sulfate is water soluble and available in both intravenous (i.v.) and oral (p.o.) formulations. Unlike the i.v. formulations of voriconazole and posaconazole, isavuconazonium sulfate does not require the addition of cyclodextrin to facilitate solubility, therefore eliminating the concerns of nephrotoxicity from this vehicle. The pharmacokinetics (PK) of isavuconazole have been assessed in healthy adult volunteers and neutropenic and immunocompromised adult patients (10–13). Attractive PK characteristics, such as slow elimination and a high volume of tissue distribution, were observed in these clinical trials. However, serum/plasma drug levels do not provide definitive information on drug-pathogen interactions at the site of infection (14–16). Currently, there is a paucity of data assessing tissue penetration of isavuconazole or other antifungal drugs at infected tissue sites.

Chronic granulomatous disease (CGD) is an inherited disorder of the NADPH oxidase complex in which phagocytes are defective in generating the reactive oxidant superoxide anion and its metabolites hydrogen peroxide, hydroxyl anion, and hypohalous acid. As a result, CGD patients suffer from recurrent life-threatening bacterial and fungal infections. Patients with CGD have the highest lifetime risk of IA (incidences of 26% to 45%), and despite the availability of antifungal prophylaxis and targeted antifungal therapy, IA remains the most common infectious complication and the most frequent cause of death in CGD patients (17). IA in CGD is characterized by a subacute infection, with nonangioinvasive, excessive granuloma formation in the affected tissue.

In this study, we investigated drug penetration of isavuconazole at the site of infection in experimental IA established in mice with CGD (gp91^phox−^). This model recapitulates human disease, and the animals are highly susceptible to *Aspergillus* infection (18, 19). Using matrix-assisted laser desorption ionization mass spectrometry imaging (MALDI-MSI) (20) and laser capture microdissection (LCM)-directed high-pressure liquid chromatography coupled to tandem mass spectrometry (LC-MS/MS) (21, 22), we visualized the spatial distribution of isavuconazole in infected lungs and brains, quantified absolute drug levels in distinct subcompartments of infected tissue, and correlated these levels with histopathological results.

## RESULTS

### Serum PK and overall distribution in lungs and brain.

**FIG 1:**
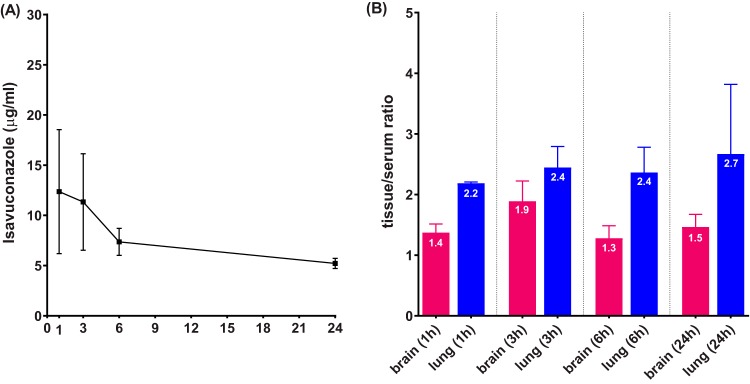
Isavuconazole serum drug concentration-time curve (A) and tissue/serum ratios (B) 1, 3, 6, and 24 h following a single oral administration of isavuconazonium sulfate at 256 mg/kg.

The serum drug concentration-time curve following a single oral dose of 256 mg/kg of body weight of the prodrug is plotted in Fig. 1A. The mean peak concentration was 12.4 μg/ml, achieved at 1 h postdose. Drug levels slowly decreased over time, and an average of 5.2 μg/ml isavuconazole was circulating at 24 h after dosing. Drug concentrations were also quantified in lung and brain tissues regardless of histopathology or anatomical structure. Tissue/serum drug ratios (Fig. 1B) demonstrated that isavuconazole followed a quick and sustained pattern to distribute into both lungs and brain. Not only were both target organs found to have drug concentrations higher than serum levels (tissue/serum ratio of >1) at each time point, but drug elimination was at least comparable with or slower than that in blood, especially in lungs (ratio slightly increased from 2.2 at 1 h to 2.7 at 24 h postdose).

### Penetration in infected lungs.

**FIG 2:**
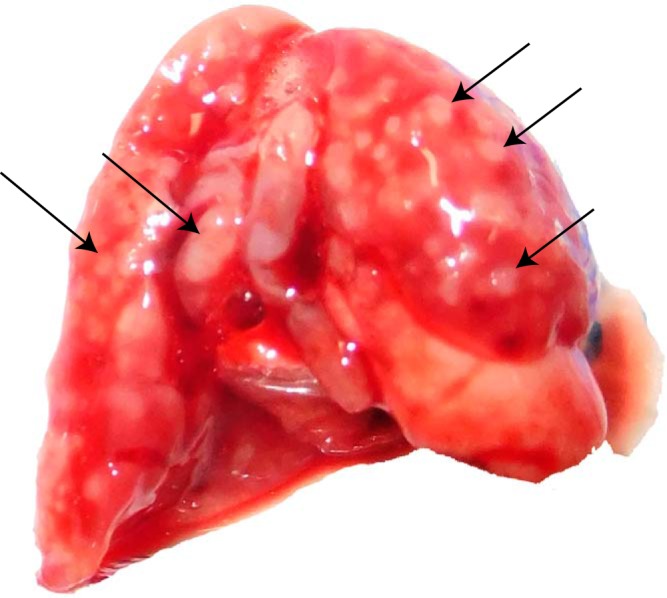
Infected mouse lung at day 7 postinoculation. Arrows point out multiple lesions formed in the lung.

**FIG 3:**
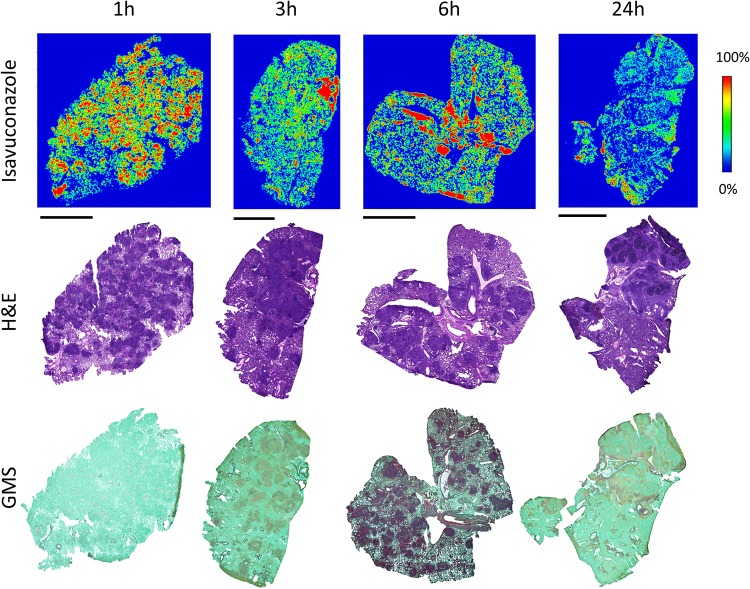
Drug distribution in infected lung tissues after a single oral dose of isavuconazonium sulfate. The top row shows ion maps of isavuconazole in representative lung tissues collected at 1, 3, 6, and 24 h postdose. The signal intensity color bar is fixed for isavuconazole, with gradually increased intensity from blue (no signal) to red (maximum signal). H&E and GMS staining of adjacent sections are shown below each ion map. Bars, 5 mm.

**FIG 4:**
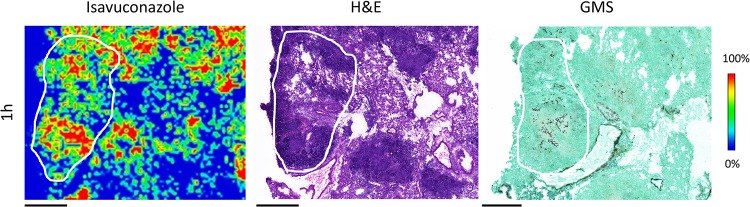
Zoomed isavuconazole image (left) at the 1-h-postdose time point showing that the hot spots of drug signals largely correlate with granuloma lesions (H&E staining of adjacent section [middle]), within which fungal hyphae aggregate (GMS stain of adjacent section [right]). Outlines highlight the lesion area on each tissue section. Bars, 1 mm.

**FIG 5:**
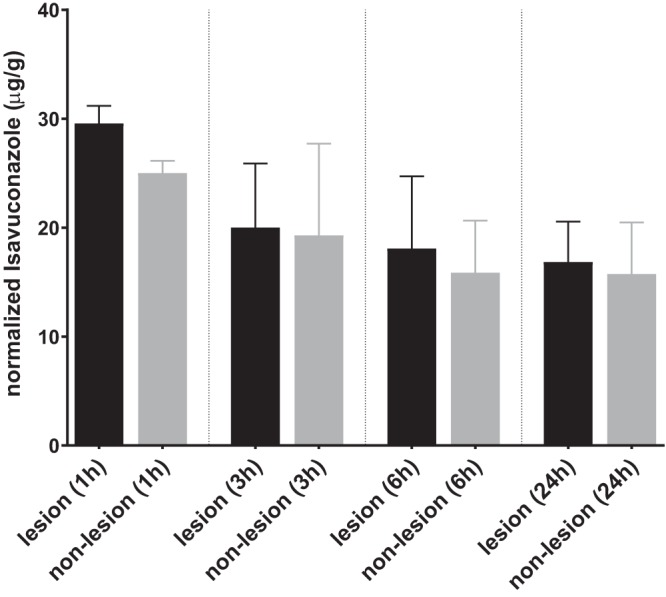
Quantification of drug exposure in lung lesions and nonlesion areas. The isavuconazole concentration was measured in lesions and nonlesion areas dissected from lung sections collected 1, 3, 6, and 24 h after a single oral dose of isavuconazonium sulfate at 256 mg/kg. Error bars show means ± standard deviations (SD) from 3 lung sections.

The development of pulmonary aspergillosis in CGD mice presented as multiple granulomatous lesions diffused in infected lungs at ∼3 to 7 days postinoculation (Fig. 2). To uncover the relationship between drug distribution, tissue histopathology, and location of invasive fungal cells, serial tissue sections were subjected to MALDI-MSI and hematoxylin and eosin (H&E) and Gomori methenamine-silver (GMS) staining. The predominant histological finding from infected but untreated lungs was granulomatous inflammation ranging from well-defined granulomas to diffused collections of neutrophil infiltrates in the center surrounded by histiocytes and lymphocytes, occasionally with necrosis. Clusters of fungal cell aggregates were largely residing within and around granulomatous lesions, while invasive hyphal elements scattering outside lesions were also observed. The spatial distribution of isavuconazole after a single oral dose was visualized by MALDI-MSI, constructing high-resolution heat maps of relative drug concentrations within targeted tissue beds (Fig. 3). Within 24 h postdose, the overall drug intensity in lung tissues peaked at 1 h postdose and slowly decreased over time, indicating the quick distribution but slow elimination of isavuconazole in infected lungs. A heterogeneous partition of isavuconazole was initially observed at 1 h postdose, as drug signal hot spots seem to correlate with the distribution of the pyogranulomatous lesions (Fig. 4). However, this “drug sticking to lesions” effect was diluted over time, and a more equilibrated distribution was observed in lungs collected at later time points. This finding was further confirmed by LCM-directed drug quantification in distinct compartments of involved lung tissues (Fig. 5). At 1 h postdose, the average drug levels were measured at 29.7 and 25.0 μg/g in lesions and nonlesion areas, respectively. Even though this drug concentration difference was not statistically significant, possibly due to the small sample size (data acquired from 3 mice with formation of lesions in lungs at this time point), drug levels were numerically more similar between lesion and nonlesion areas at later time points. The isavuconazole concentrations were measured as 20.0 versus 19.3, 18.1 versus 15.9, and 16.8 versus 15.8 μg/g in lesions versus nonlesions at 3, 6, and 24 h postdose, respectively.

**FIG 6:**
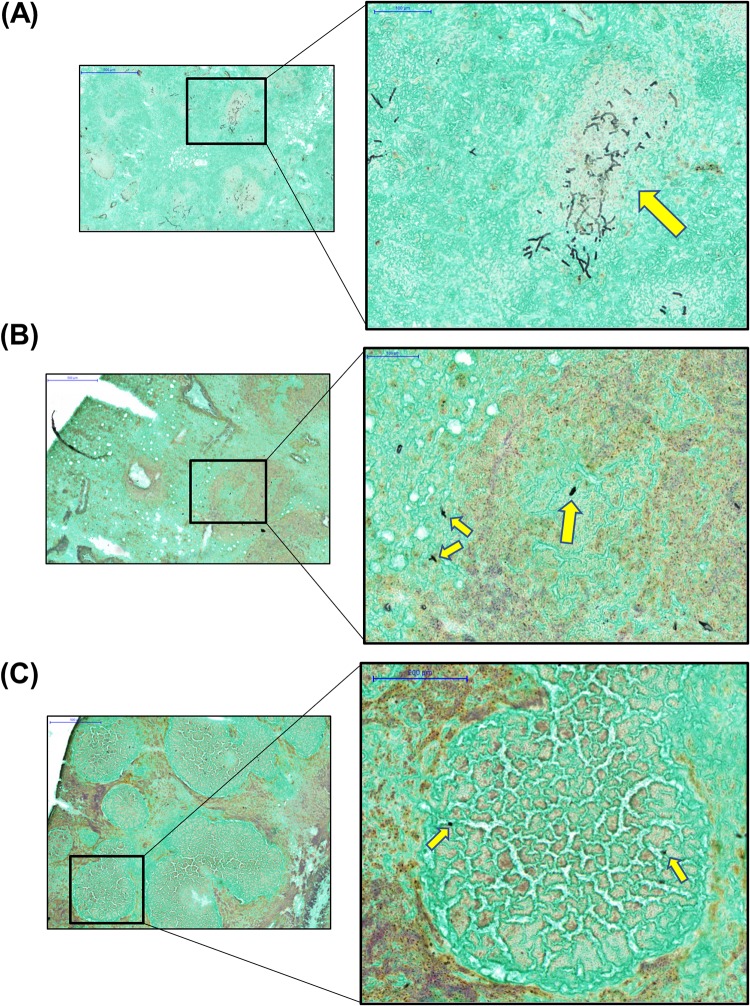
Representative GMS-stained lung sections at 1 h (A), 3 h (B), and 24 h (C) postdose. Yellow arrows in the enlarged views (right [magnification, ×20]) of black boxed areas (left [magnification, ×5]) at each time point indicate fungal cells.

In an attempt to explore the spatial relationship between the drug and fungal cells, an unexpected, rapid fungal growth inhibition effect of this high dosage of isavuconazole was revealed. Specifically, branched hypha clusters residing in or around granulomatous lesions were observed only at 1 h postdose, and remarkably fewer and smaller hyphal elements scattered in and outside lesions presented in all sections collected at 3 h postdose (Fig. 6). At 24 h postdose, a further reduced number of rounded fungal cells was observed (Fig. 6), although the granulomatous inflammation was not much different from what was observed at earlier time points.

### Penetration in infected brains.

**FIG 7:**
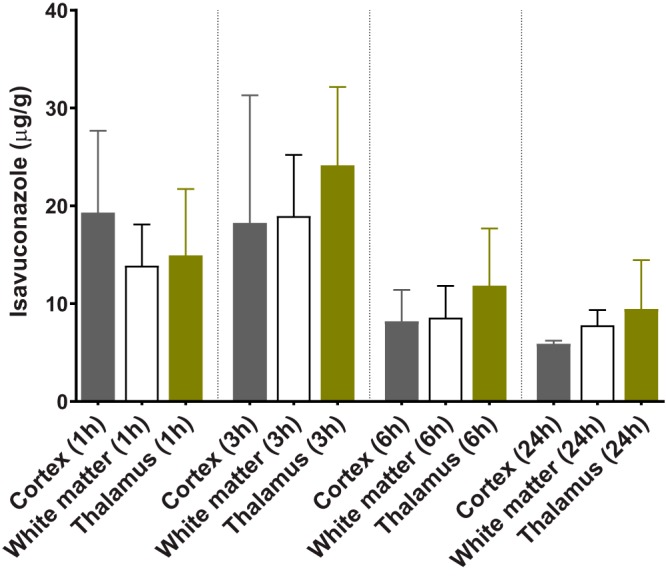
Quantification of isavuconazole in brain compartments at post-single-dosing time points.

Brains collected from infected CGD mice did not show noticeable gross abnormality, although histopathology showed fungal element involvement (see Fig. S1 in the supplemental material). Examination of H&E-stained brain sections showed disseminated neutrophilic inflammation without clear formation of granulomas. An isavuconazole ion map captured at 1 h postdose (Fig. S2) displayed efficient penetration and a relatively homogeneous drug distribution pattern in the infected brain. In light of the absence of visible brain lesions, we dissected brain tissues based on the anatomical structure and measured drug levels in cortex, white matter, and thalamus at each time point. Consistent with the imaging results, drug concentrations were not significantly different in these three compartments at all time points (Fig. 7). Peak levels were acquired at 3 h postdose in both white matter and thalamus, at 19.0 and 24.1 μg/g of drug, respectively, although the maximum concentration in cortex appeared earlier, 1 h after dosing. Overall, brain had delayed peak exposure relative to blood and lungs, and drug retention was higher in thalamus than in cortex and white matter.

## DISCUSSION

As the new member of the triazole antifungal class, isavuconazole (the active moiety of the prodrug isavuconazonium sulfate) has demonstrated potent activity against a wide spectrum of fungal pathogens in a number of studies both *in vitro* and *in vivo* (8, 9, 23–27). As a result, it has been approved by the U.S. Food and Drug Administration (FDA) for the treatment of invasive aspergillosis and mucormycosis. Although isavuconazonium sulfate has demonstrated clinical efficacy, and there are a few case reports of successful treatment of cerebral infections (28, 29), we generally lack a full appreciation of how isavuconazole distributes and penetrates at infected tissue sites, except for the limited data acquired in healthy animals by quantitative whole-body autoradiography (QWBA) (30) and drug concentrations determined in *Candida*-infected mouse kidney homogenates (31). In view of the fact that drug penetration at the site of infection is critical to efficacy and a better understanding of this topic is of clinical significance, we employed advanced analytical tools and provided, for the first time, detailed information on drug distribution and penetration in infected tissue sites, using a CGD mouse model of pulmonary aspergillosis.

Considering the relatively short half-life of isavuconazole in mice (23) compared to that in humans and the intent to quantify drug exposure at later time points after a single dose, we used isavuconazole at a dose higher than the clinically used dose (based on the area under the curve from 0 to 24 h [AUC_0–24_] reported from population PK analysis in phase 1 and phase 3 trials as well as mouse experimental PK studies [23, 32], the approximate humanized dose in a mouse is 160 mg/kg of prodrug). The serum isavuconazole concentration-time profile obtained in this study was consistent with data reported previously (23). By measuring overall drug levels in lungs and brain and calculating tissue/serum ratios, a pattern of rapid and efficient distribution was observed with isavuconazole in these two important target organs of infections, confirming previous findings acquired by QWBA (30). A more in-depth examination at the infection sites revealed that not only is isavuconazole quickly distributed into lung tissue after oral administration, but the drug seems to be transiently trapped in granulomatous lesions compared to the nonlesion area once partitioned into the lungs. This early-phase higher drug level in the lesion pockets is possibly due to the large number of inflammatory cells forming pyogranulomas, as fungal cells trapped inside are more apt to take up isavuconazole than are alveolar cells in the regions yet to be involved in the exuberant inflammatory reaction to fungal invasion. With the overall tissue drug levels decreasing over time, isavuconazole equilibrated between lesion and nonlesion areas from 3 h to 24 h postdose, indicating that drug partitioning was quickly balanced within infected lungs and that drug retention was not that different in various subcompartments. Nevertheless, the underlying mechanism of this particular kinetic pattern of tissue distribution warrants further studies. Although this study was not intended to evaluate efficacy, it is intriguing that a rather quick and remarkable antifungal activity was observed in the exploration of locating fungal cells within infected lung tissues. This drastic growth inhibition effect, notwithstanding its identification by a semiquantifiable method, is presumably a result of the drug exposure (∼30 μg/g) largely exceeding the required concentration for inhibition acquired at early time points postdose in the granulomatous lesions, where the majority of the fungal population resides. It should be noted that the use of a susceptible strain (isavuconazole MIC of 0.25 μg/ml for R21) also contributed to the rapid eradication effect observed in our study. Given that the AUC/MIC ratio is the pharmacodynamic (PD) driver for isavuconazole (23), an increase of the MIC (reduced susceptibility) for the infecting strain will negatively affect the antifungal efficacy of isavuconazole, similar to what was reported in a previous mouse PK/PD study (26).

Due to the lack of obvious pathology in brain, we analyzed the drug distribution based on the anatomical structure. In general, brain had a slightly delayed drug concentration peak compared to blood and lungs. No significant drug level difference was observed in the three compartments analyzed, although thalamus seemed to have the highest drug retention relative to cortex and white matter. The absolute drug concentrations in all assessed brain compartments were sufficiently high to inhibit fungal growth, in keeping with previous QWBA results (30) and supporting the use of isavuconazole in the treatment of cerebral fungal infections.

There are a few limitations of this study. First, we used CGD mice as our working model to demonstrate drug penetration. Even though CGD is clinically important and common, it is not a universal condition in the entire IA patient population. Often, non-CGD IA patients are neutropenic and hence have different histopathology as well as host response to drug exposure compared to those with underlying CGD. Therefore, the conclusions of this study are largely limited to CGD patients. Second, investigation of drug penetration was limited to single-dose administration, and a repeated-dose experiment was not performed. It is likely that dose-dependent penetration may occur, and the penetration pattern observed with the current dose may not be present at all doses. However, as the first study exploring triazole drug penetration at the site of infection, it establishes a working model for other triazole antifungals and provides data of clinical relevance. Third, the nonlesion area in infected lung tissues may not truly be uninvolved in the massive inflammation triggered by pulmonary *Aspergillus* infection in CGD mice. Given that infected lungs contained numerous and diffused coalescing granulomas and that inflammatory cells and fungal cells still scattered outside granulomatous lesions, drug quantification in nonlesion areas may be affected by such a drug-loaded cell population included in the dissection. Finally, except for the semiquantified fungal load evaluation as part of histopathological analysis, an antifungal effect was not evaluated and correlated with drug exposure in our study. A more comprehensive study regarding the dose-response relationship at the site of infection is warranted.

In summary, our study demonstrates that isavuconazole penetrates into *Aspergillus*-infected lungs and brain highly efficiently. The drug level in tissue compartments in which fungi reside was sufficiently high to inhibit fungal growth and reduce fungal burdens in these organs. These data further support the use of isavuconazonium sulfate to treat CGD patients with invasive aspergillosis.

## MATERIALS AND METHODS

### Antifungal agents.

Isavuconazole (BAL4815) and the prodrug isavuconazonium sulfate (BAL8557) were provided by Astellas Pharma Global Development. For dosing, the prodrug was dissolved in sterile water prior to oral administration. The conversion factor for determining the equivalent isavuconazole dose from the prodrug dose was 0.48 on a milligram-per-kilogram basis (provided by Astellas).

### Strain.

Aspergillus fumigatus strain R21 (33) was grown on potato dextrose agar (PDA) slants for 3 days at 37°C. Spores were harvested, washed, and suspended in sterile saline containing 0.01% Tween 20 to a final concentration of 1 × 10^5^ spores/ml for infection.

### Experimental animals.

Male 9-week-old CGD mice (gp91^phox−^) (The Jackson Laboratory, Bar Harbor, ME, USA) were housed in the Public Health Research Institute’s Animal Biosafety Level 2 Research Animal Facility (ICPH RAF), a center of the New Jersey Medical School, Rutgers University (NJMS-Rutgers). All experimental procedures were performed in accordance with National Research Council guidelines (34) and approved by the Rutgers University Research Institutional Animal Care and Use Committee (IACUC).

### Experimental IA in CGD mice, isavuconazole treatment, and sample collection.

Mice were infected with 1 × 10^5^ spores of A. fumigatus R21 in 25 μl of a conidial suspension via intratracheal (i.t.) administration, as previously described (35). On day 4 postinoculation or when weight loss reached 15%, a single oral (p.o.) dose of isavuconazonium sulfate (256 mg/kg, corresponding to 122.9 mg/kg of isavuconazole) or the vehicle control was administered to the mice. Mice were sacrificed immediately prior to treatment (*n* = 1) and at 1, 3, 6, and 24 h postdose (5 mice per time point). Lungs and brains were explored for abscesses, dissected, placed on a cryohistology tray, snap-frozen in liquid nitrogen, and stored at −80°C for MALDI-MSI and LCM-directed drug quantification. Blood was also collected at each time point for serum drug concentration measurement.

### Tissue sectioning.

Using a Leica (Buffalo Grove, IL) CM1860 UV cryostat, 12-μm-thick tissue sections were mounted onto stainless steel slides for MALDI-MSI analysis and frosted glass microscope slides for GMS and H&E staining (21). Adjacent 25-μm-thick tissue sections were mounted onto a thin polymer membrane slide for laser capture microdissection. All slides were air dried for 10 min, sealed in a small airtight sealable bag, and stored at −80°C until analysis.

### Matrix application and rehydration chamber method.

Using an HTX (Chapel Hill, NC) TM-sprayer operating with a 50-μl/min flow rate, a 60°C nozzle temperature, and 5 lb/in^2^ of pressure, 2,5-dihydroxybenzoic acid (DHB) (25 mg/ml in 50% methanol [MeOH]) containing 50 μl of an internal standard (1 mg of deuterated isavuconazole dissolved in 1 ml of 100% methanol) was applied to the surface. Twenty-five passes over the tissue were performed. Immediately after spraying the matrix, a matrix recrystallization method was followed to increase drug sensitivity. A small cloth was placed on the lid of a petri dish and wetted with 500 μl of water. Matrix-coated tissue slides for MALDI-MSI analysis were taped on the bottom of the petri dish. The petri dish was sealed and incubated for 7 min at 37°C.

### MALDI-MSI analysis.

MALDI-MSI analysis was performed using a MALDI LTQ Orbitrap XL mass spectrometer (Thermo Fisher Scientific, Bremen, Germany) with a resolution of 60,000 at *m/z* 400, with full width at half-maximum (20). The resolution was sufficient to resolve isavuconazole peaks from the background without the requirement for MS/MS and the subsequent loss of signal. However, drug peak identities were confirmed by acquiring several MS/MS spectra directly from the dosed tissues. Standards of isavuconazole were analyzed directly from the stainless steel target plate and spiked into drug-naive lung tissue to optimize instrument parameters. The limit of detection (LOD) for MALDI-MSI analysis of isavuconazole was 5 μg/g of lung or brain tissue, calculated as described previously (14). Spectra were acquired in the *m/z* 420 to 460 range, using the positive ionization mode. Data visualization was performed using Thermo ImageQuest software. Normalized ion images of isavuconazole were generated by dividing the isavuconazole [M + H]^+^ signal (*m/z* 438.118 ± 0.005) by the isavuconazole-d5 [M + H]^+^ signal (*m/z* 443.146 ± 0.005).

### Laser capture microdissection.

Distinct subcompartments of infected lung tissue (e.g., lesion and surrounding uninvolved tissue) totaling 1 million to 3 million μm^2^ were carefully dissected from between 4 and 6 serial lung tissue sections using a Leica (Buffalo Grove, IL) LMD 6 (CC7000) system (22). Cortex, white matter, and thalamus areas were also dissected from brain tissue sections. Lesion areas were identified optically from the bright-field image scan and by comparison to the adjacent sectioned GMS- and H&E-stained tissue. The dissected tissues were collected into 0.25-ml standard PCR tubes and immediately stored in an −80°C freezer unless analyzed immediately. The PCR tubes were thawed at room temperature prior to analysis and then centrifuged for 5 min at 4,000 rpm to sediment the laser-captured tissues to the bottom of the tube. Fifty microliters of an extraction solution (1:1 mixture of acetonitrile [ACN]-MeOH containing 10 ng/ml verapamil internal standard), 10 μl of a 1:1 mixture of an ACN-H_2_O solution, and 2 μl of phosphate-buffered saline (PBS) were added to each tube containing microdissected tissues, which were then sonicated for 10 min and centrifuged at 4,000 rpm for 5 min at room temperature. Standard curve and quality control tubes were created by combining 50 μl of extraction solution, 10 μl of serially diluted isavuconazole in ACN-H_2_O, and 2 μl of lung or brain homogenate from untreated animals (22), followed by sonication and centrifugation as described above for study samples. Fifty microliters of the supernatant was transferred from both study samples and standards for LC-MS/MS analysis and diluted with an additional 50 μl of deionized water in a 96-well deep-well plate.

### Drug quantitation by LC-MS/MS.

LC-MS analysis was performed on an AB Sciex (Ontario, Canada) Qtrap 6500^+^ instrument coupled to a Shimadzu HPLC system. Chromatography was performed with an Agilent SB-C_8_ 2.1- by 30-mm, 3.5-μm-particle-size column using a reverse-phase gradient. Mobile phase A was 0.1% formic acid in 100% water, and mobile phase B was 0.1% formic acid in 100% ACN. Drug quantitation in tissue was conducted using transitions from *m*/*z* 438.06 to 224.00 for isavuconazole and *m*/*z* 455.40 to 165.20 for verapamil, which was used as an internal standard.

### Statistical analysis.

Absolute drug concentrations were graphed and statistically analyzed using GraphPad software (Prism 7; GraphPad Software, Inc., San Diego, CA). Drug levels in different tissue compartments at various time points were compared by one-way analysis of variance (ANOVA), and Dunn’s multiple-comparison test was used for *post hoc* analyses. Statistical significance was defined as a *P* value of <0.05.

## Supplementary Material

Supplemental file 1
