# Supplementary material for: Tissue Distribution and Penetration of Isavuconazole at the Site of Infection in Experimental Invasive Aspergillosis in Mice with Underlying Chronic Granulomatous Disease
Source: Antimicrob Agents Chemother. 2019 May 23;63(6):e00524-19. doi: 10.1128/AAC.00524-19 (PMC6535567; doi:10.1128/AAC.00524-19)
Supplement: Supplemental file 1 [file AAC.00524-19-s0001.pdf]

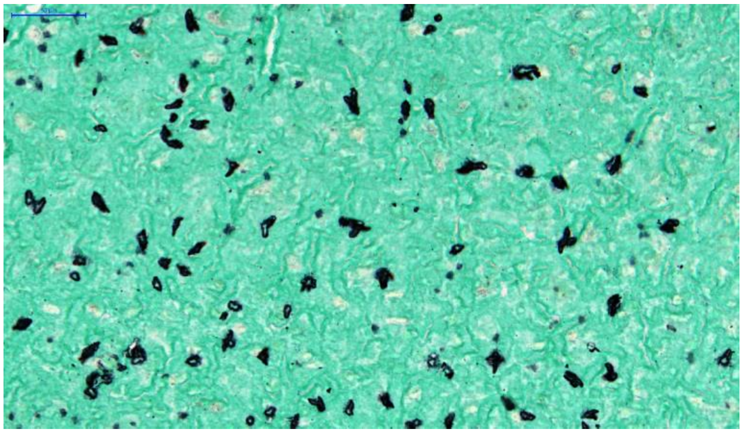

Supplemental Figure 1. Histopathology showing fungal elements in the infected brain (GMS stain, 40×)

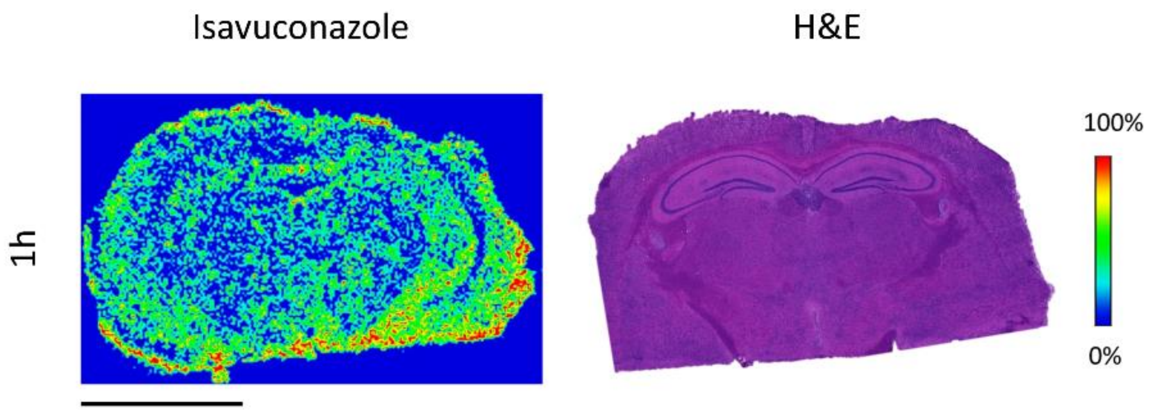

Supplemental Figure 2. Drug distribution in infected brain at 1 h after a single oral dose of isavuconazonium sulfate at 256 mg/kg. The signal intensity color bar is fixed for isavuconazole, with gradually increased intensity from blue (no signal) to red (max signal). H&E (right) staining of adjacent section is shown. Scale bar, 5 mm.
